# A Challenging Case of Secondary Hyperparathyroidism From Hypovitaminosis D in a Young Man With Hypertensive Crisis and Target Organ Damage

**DOI:** 10.7759/cureus.94596

**Published:** 2025-10-14

**Authors:** Abdelrahim Elmejrab, Zain Al Abdeen Al Zuabi

**Affiliations:** 1 General Medicine, The Queen Elizabeth Hospital King's Lynn NHS Foundation Trust, King's Lynn, GBR; 2 Orthopaedics, The Queen Elizabeth Hospital King's Lynn NHS Foundation Trust, King's Lynn, GBR

**Keywords:** hypertensive crises, hypovitaminosis d, rare case report, secondary hyperparathyroidism, secondary hypertension

## Abstract

Hypertension is a common global condition, with most cases being primary and a minority arising from secondary causes, often endocrine in origin. Young patients presenting with hypertensive crises are more likely to have secondary aetiologies, necessitating a systematic and comprehensive workup. This case report describes a 37-year-old man presenting with severe headache, visual disturbances, and chest pain, with a blood pressure of 250/170 mmHg. Evaluation revealed advanced retinopathy, cerebral microhaemorrhages, renal impairment, and left ventricular hypertrophy, consistent with target organ damage. Further investigations identified secondary hyperparathyroidism due to profound hypovitaminosis D as the likely cause. Targeted treatment corrected the vitamin D deficiency and hypocalcaemia while gradually controlling blood pressure, leading to a favourable outcome. This exceptionally rare presentation underscores the need for wide-ranging diagnostic assessment in young patients with hypertensive crises, as identifying reversible secondary causes can significantly influence prognosis and long-term morbidity.

## Introduction

Hypertension is a leading global health challenge, with most cases classified as primary and only a minority arising from secondary causes [1]. Nevertheless, in young patients presenting with hypertensive crises, the possibility of a reversible secondary aetiology becomes critical [2]. Hypertensive crisis is an umbrella term used when blood pressure exceeds 180/120 mmHg. The term hypertensive emergency is used when there is evidence of acute target organ damage, such as to the brain, heart, kidneys, or eyes, demanding urgent stabilization alongside a systematic diagnostic evaluation to uncover hidden causes. When blood pressure is similarly elevated but no acute target organ damage is present, it is termed a hypertensive urgency [3]. This case report describes a 37-year-old man with hypertensive emergency and widespread organ involvement, in whom an unexpected culprit, secondary hyperparathyroidism from severe hypovitaminosis D, was identified. This rare association highlights the importance of maintaining diagnostic vigilance, as recognizing and correcting uncommon but reversible causes can profoundly alter the clinical trajectory.

This article was previously presented as a poster at the 6th International Conference of the European Society of Cardiology Council on Stroke held on December 2 and 3, 2023, in Oxford, United Kingdom.

## Case presentation

A 37-year-old man presented to the emergency department with severe headache, visual disturbances, chest pain, and dizziness. On admission, he was fully alert (Glasgow Coma Scale 15) with a blood pressure of 250/170 mmHg measured in both right and left arms, a heart rate of 96 beats/minute, a respiratory rate of 16 breaths/minute, a temperature of 37°C, and an oxygen saturation of 99% on room air. The headache, rated 6/10 in severity, was localized in the occipital region, accompanied by blurred vision that had developed on the same day. He also experienced several transient episodes of near syncope. Neurological and cardiopulmonary examinations were unremarkable. His medical history was unremarkable, with no prior hospitalizations, medications, allergies, or substance use. Notably, his brother had died suddenly at a young age from an unexplained cause.

**Figure 1:**
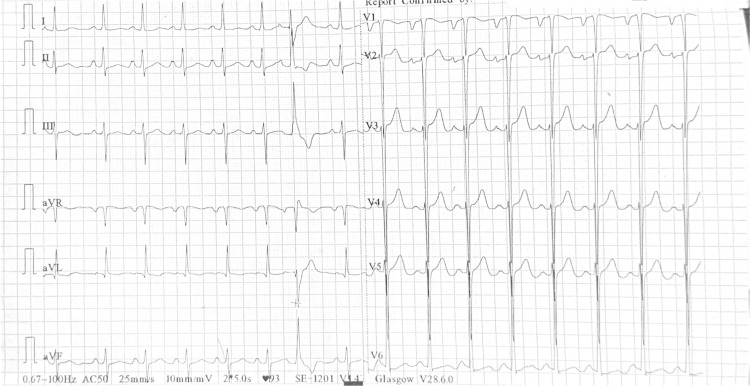
Twelve-lead ECG at initial presentation showing sinus rhythm with a heart rate of 93 bpm, left ventricular hypertrophy by Sokolow-Lyon criteria, P mitrale, and a prolonged QTc interval of 476 ms ECG: electrocardiogram

**Figure 2:**
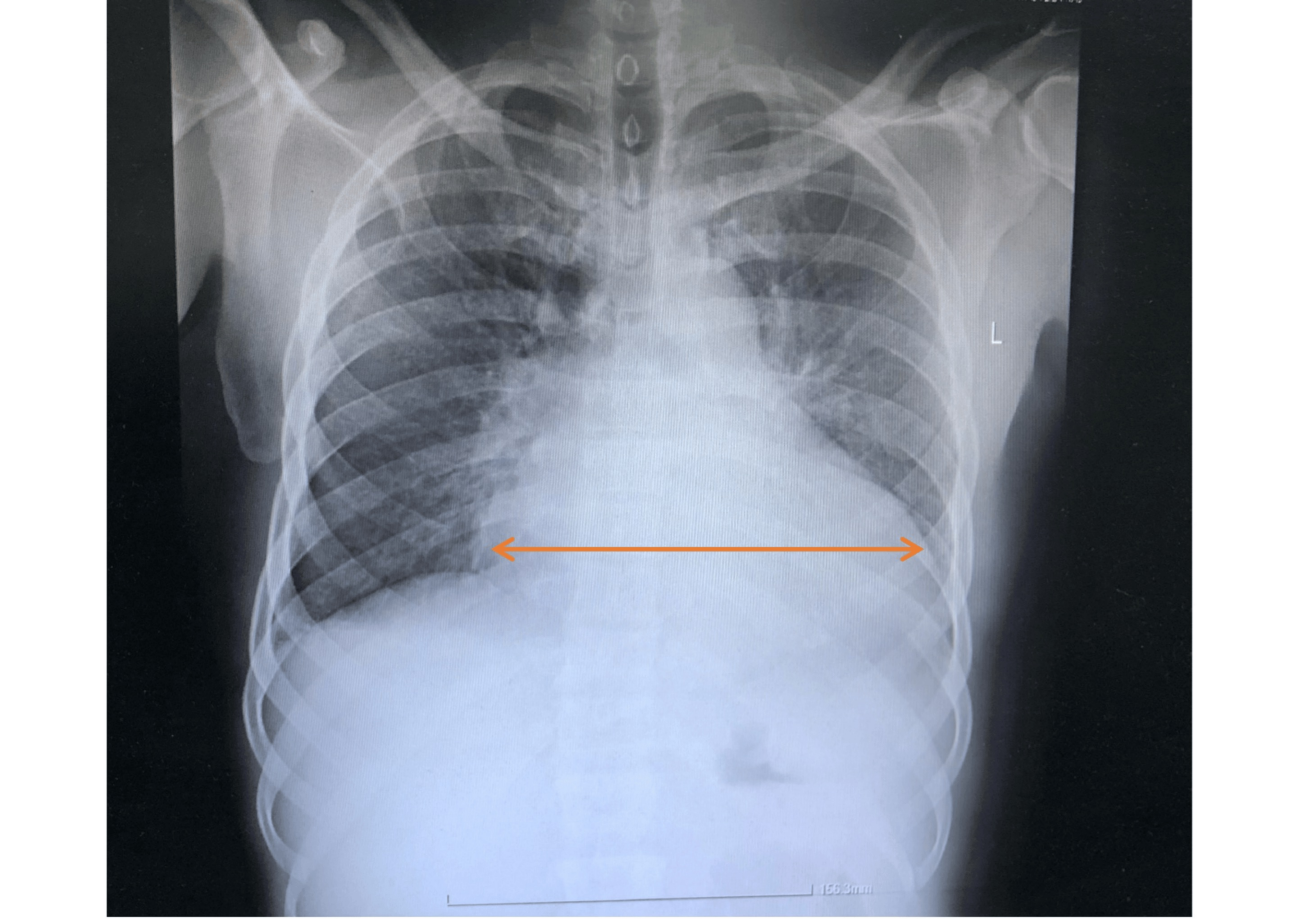
Erect chest X-ray showing cardiomegaly with a cardiothoracic ratio of 0.75 (arrow)

Initial evaluation confirmed malignant hypertension with features of hypertension-mediated organ damage. An electrocardiogram (ECG) demonstrated sinus rhythm with left ventricular hypertrophy, P mitrale, and a prolonged QTc of 476 ms (Figure 1). Chest X-ray showed an increased cardiothoracic ratio (Figure 2). Transthoracic echocardiography revealed left atrial and left ventricular enlargement, preserved systolic function, and mild mitral regurgitation. Renal function was impaired (urea 18.6 mmol/L; normal 2.5-7.8 mmol/L; creatinine 187 µmol/L; normal 60-110 µmol/L), while other biochemical parameters were within normal limits. Serial troponins were negative. Ophthalmic examination revealed hypertensive retinopathy. Brain computed tomography (CT) demonstrated multiple cerebral microhaemorrhages, consistent with hypertensive end-organ injury (Video 1).

**Video 1:** CT of the head showing multiple cerebral microhaemorrhages secondary to hypertension crisis

**Figure 3:**
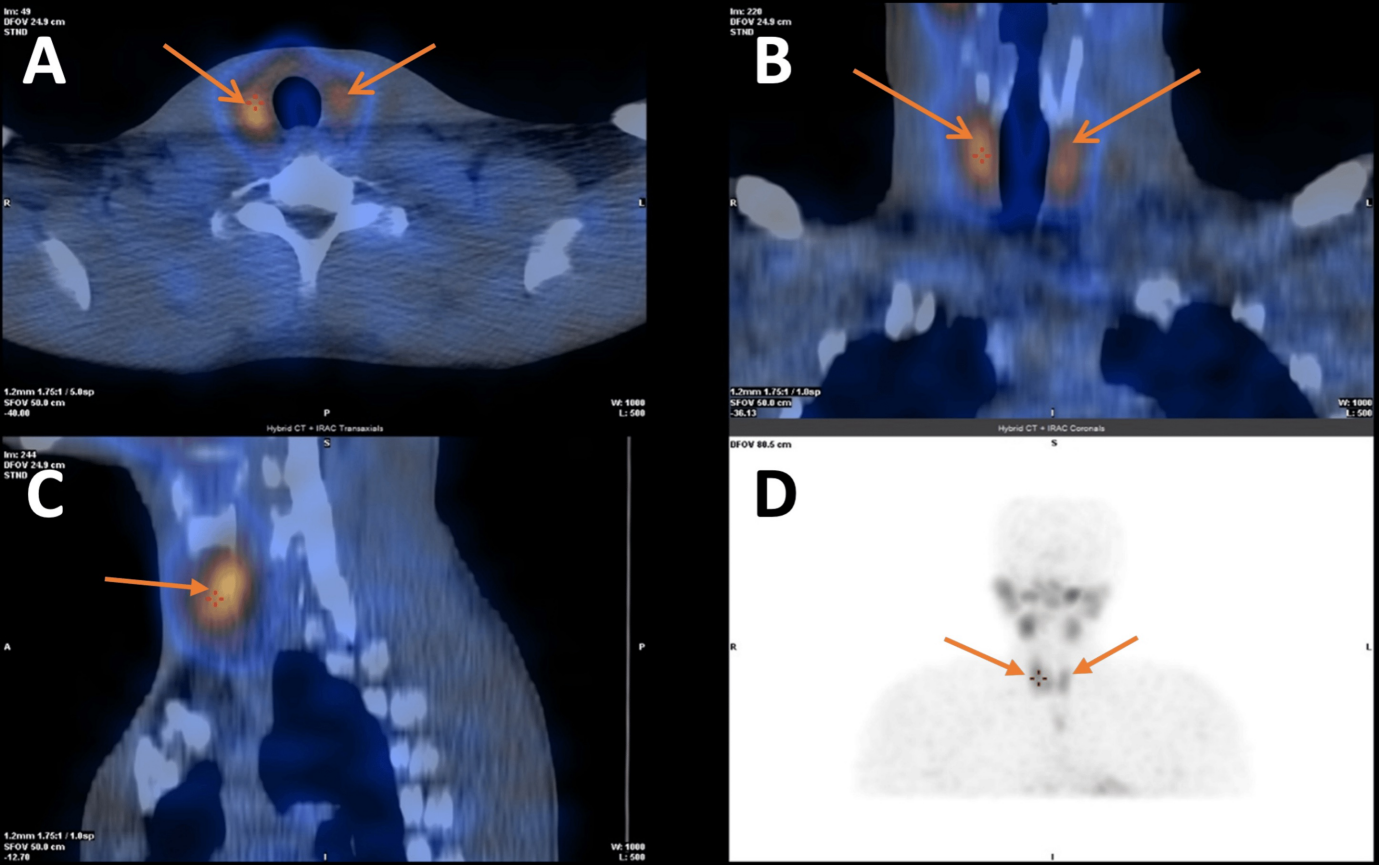
Parathyroid dual isotope (I-123 and Tc-99m-sestamibi) planar pinhole subtraction scintigraphy with subtraction SPECT/CT demonstrating reactive secondary hyperparathyroidism and excluding a primary parathyroid adenoma (A) Axial dual isotope subtraction SPECT/CT showing reactive parathyroid glands bilaterally with the right glands being more reactive than the left (arrows). (B) Coronal dual isotope subtraction SPECT/CT showing reactive parathyroid glands bilaterally with the right glands being more reactive than the left (arrows). (C) Sagittal dual isotope subtraction SPECT/CT showing a reactive parathyroid gland (arrows). (D) Coronal dual isotope planar pinhole subtraction scintigraphy showing reactive parathyroid glands bilaterally with the right glands being more reactive than the left (arrows) SPECT/CT: single-photon emission computed tomography/computed tomography

Given his age, presentation with a hypertensive emergency, and widespread organ involvement, secondary hypertension was strongly suspected. Common causes, including renal disease, aortic coarctation, obstructive sleep apnoea, primary hyperaldosteronism, hyperthyroidism, and pheochromocytoma, were systematically excluded. Biochemical analysis revealed markedly elevated parathyroid hormone (192.5 pg/mL; normal 15-65 pg/mL) with hypocalcaemia (2.0 mmol/L; normal 2.2-2.6 mmol/L) and profound vitamin D deficiency (20 nmol/L; sufficient >50 nmol/L), consistent with secondary hyperparathyroidism. Parathyroid dual isotope (I-123 and Tc-99m-sestamibi) planar pinhole subtraction scintigraphy with subtraction single-photon emission computed tomography (SPECT)/CT was performed to further evaluate parathyroid activity and exclude primary adenoma (Figure 3).

Acute blood pressure management involved intravenous labetalol and nicardipine, targeting a 25% reduction in mean arterial pressure, followed by oral hydrochlorothiazide and amlodipine. Vitamin D and calcium supplementation were initiated concurrently. Blood pressure gradually stabilized, visual symptoms and dizziness improved, renal function recovered, and the QTc interval normalized. Investigations into the cause of vitamin D deficiency suggested inadequate sunlight exposure and dietary intake. The patient was discharged without major complications and continues under regular outpatient follow-up with satisfactory progress.

## Discussion

Hypertension in young adults, particularly when presenting as a hypertensive crisis or with evidence of target organ damage, warrants a heightened suspicion for secondary causes. Unlike primary hypertension, which predominates in the general population, secondary forms are often reversible if identified early [4]. This case underscores the importance of a systematic and comprehensive diagnostic approach. Clinicians must undertake meticulous history-taking, thorough physical examination, and strategic laboratory and imaging investigations to uncover end-organ dysfunction, including subclinical involvement of the heart, kidneys, eyes, and brain. In patients presenting with markedly elevated blood pressure, a careful assessment for hypertension-mediated organ damage is not only informative for diagnosis but also crucial for guiding safe management [5]. Even when common secondary causes are excluded, clinicians should maintain vigilance for rarer aetiologies, as timely recognition can profoundly alter patient outcomes.

The association between secondary hyperparathyroidism triggered by hypocalcaemia and hypertension remains sparsely described in the literature. The pathophysiology is incompletely understood, though it is hypothesized that elevated circulating parathyroid hormone may induce endothelial dysfunction, contributing to increased vascular tone and blood pressure elevation [6,7]. Our case demonstrates this rare but clinically significant link and highlights the need for further investigation into the interplay between vitamin D deficiency, hypocalcaemia, elevated parathyroid hormone levels, and hypertension. Large-scale studies are required to better elucidate these mechanisms and to define the potential for targeted interventions in affected patients [8,9]. Importantly, this case exemplifies how a seemingly complex hypertensive crisis can sometimes have a straightforward and treatable underlying cause, as in this patient where calcium and vitamin D supplementation alone corrected the biochemical abnormalities and contributed to blood pressure control.

Management of hypertensive emergencies requires caution, as overly rapid reduction of blood pressure can precipitate cerebral, cardiac, or renal hypoperfusion, compounding organ injury. In addition to careful pharmacologic control, the identification and treatment of the underlying cause of secondary hypertension are essential components of therapy [10]. This case also illustrates the synergistic nature of modern internal medicine, where collaboration across specialties, particularly cardiology, nephrology, and endocrinology, allows for the comprehensive evaluation and tailored management of complex presentations. By integrating multidisciplinary expertise, clinicians cannot only stabilize patients acutely but also address the root cause of hypertension, thereby preventing recurrence and long-term complications.

## Conclusions

This case report describes a 37-year-old man presenting with hypertensive crisis and multi-organ involvement, in whom secondary hyperparathyroidism from profound vitamin D deficiency was identified as the likely trigger. Although uncommon, the association between elevated parathyroid hormone, hypocalcaemia, and hypertension has been described, and this case reinforces the need to consider even rare aetiologies when evaluating young patients with hypertensive crises and target organ damage. Careful, stepwise blood pressure reduction alongside correction of vitamin D deficiency and hypocalcaemia resulted in a favourable outcome. The key learning point is the importance of maintaining a high index of suspicion for secondary hypertension in younger patients, supported by thorough clinical assessment and systematic investigation. Equally, safe management of hypertensive emergencies requires avoiding precipitous reductions in blood pressure while addressing reversible underlying causes. This case also exemplifies the value of multidisciplinary collaboration in unravelling and managing complex presentations.
